# CB2 regulates oxidative stress and osteoclastogenesis through NOX1-dependent signaling pathway in titanium particle-induced osteolysis

**DOI:** 10.1038/s41420-023-01761-y

**Published:** 2023-12-16

**Authors:** Huaqiang Tao, Xueyan Li, Miao Chu, Qiufei Wang, Ping Li, Qibin Han, Kai Chen, Pengfei Zhu, Yuefeng Hao, Xing Yang, Dechun Geng, Ye Gu

**Affiliations:** 1 Department of Orthopedics, The First Affiliated Hospital of Soochow University, No. 188 Shizi Street, Suzhou, Jiangsu China; 2 Anesthesiology department, Suzhou Municipal Hospital, Nanjing Medical University Affiliated Suzhou Hospital, 242, Guangji Road, Suzhou, Jiangsu China; 3 Department of Orthopedics, Changshu Hospital Affiliated to Soochow University, First People’s Hospital of Changshu City, the First Affiliated Hospital of Soochow University, Suzhou, Jiangsu China; 4 Department of Central Laboratory, Nanjing Medical University Affiliated Suzhou Hospital, Gusu School, Suzhou, Jiangsu China; 5 Orthopedics and Sports Medicine Center, Suzhou Municipal Hospital, Nanjing Medical University Affiliated Suzhou Hospital, 242, Guangji Road, Suzhou, Jiangsu China

**Keywords:** Stress signalling, Mechanisms of disease

## Abstract

Periprosthetic osteolysis (PPO) induced by wear particles at the interface between the prosthesis and bone is a crucial issue of periprosthetic bone loss and implant failure. After wear and tear, granular material accumulates around the joint prosthesis, causing a chronic inflammatory response, progressive osteoclast activation and eventual loosening of the prosthesis. Although many studies have been conducted to address bone loss after joint replacement surgeries, they have not fully addressed these issues. Focusing on osteoclast activation induced by particles has important theoretical implications. Cannabinoid type II receptor (CB2) is a seven-transmembrane receptor that is predominantly distributed in the human immune system and has been revealed to be highly expressed in bone-associated cells. Previous studies have shown that modulation of CB2 has a positive effect on bone metabolism. However, the exact mechanism has not yet been elucidated. In our experiments, we found that NOX1-mediated ROS accumulation was involved in titanium particle-stimulated osteoclast differentiation. Furthermore, we confirmed that CB2 blockade alleviated titanium particle-stimulated osteoclast activation by inhibiting the NOX1-mediated oxidative stress pathway. In animal experiments, downregulation of CB2 alleviated the occurrence of titanium particle-induced cranial osteolysis by inhibiting osteoclasts and scavenging intracellular ROS. Collectively, our results suggest that CB2 blockade may be an attractive and promising therapeutic scheme for particle-stimulated osteoclast differentiation and preventing PPO.

## Introduction

Total joint arthroplasty (TJA) is currently an important treatment for various primary or secondary end-stage osteoarthritis, rheumatoid arthritis, and other advanced hip and knee lesions [1–3]. In terms of the long-term results of the procedure, the 10-year utilization rate of the prosthesis has exceeded 90%. However, many studies have recently found that there is a dramatic loss of periprosthetic bone mass in the initial phase after TJA and that the decrease in bone mineral density (BMD) persists for a considerable period of time [4, 5]. In total hip arthroplasty, both femoral and acetabular sides experience varying degrees of bone loss within 1 year after surgery, with serious implications for the long-term stability and bone strength of the prosthesis [6]. Once the fracture is caused by bone loss in the long term, revision surgery is difficult and costly, which will again cause great burden and pain to the patient. Thus, reducing periprosthetic bone loss after joint replacement is crucial to prolong the use of the prosthesis and prevent periprosthetic fractures.

Previous studies have indicated that the build-up of wear particles near the prosthesis and their stimulation of heightened osteoclast activity in the periprosthetic tissue are significant factors in the advancement of PPO [7, 8]. The accumulation of wear particles stimulates a variety of cells in the surrounding tissues to express large amounts of cytokines and chemokines in an autocrine or paracrine manner, exacerbating local chronic inflammation in the prosthesis and promoting the formation of osteoclasts, ultimately causing structural disorders at the reconstructed prosthesis-bone interface [9]. As a result of the first generation or early recipients of joint replacement, many hip and knee replacement prostheses were made of stainless steel and titanium (Ti) [10]. Joint prostheses, such as tibial plateau brackets for the knee and acetabular outer cups for the hip, have been utilized for a considerable period of time. However, these prostheses have now reached the end of their useful life, necessitating revision surgery for this particular group of patients. Considering that the osteolysis model induced by Ti particles is also recognized as the most common model [11], the study of whether Ti particles have an effect on bone metabolism and the possible mechanism of action remains a topic of interest.

Our country used to use cannabis for medicinal treatment, and the main component of cannabis, tetrahydrocannabinol, acts mainly on cannabinoid receptors [12, 13]. There are two main types of cannabinoid receptors, CB1R and CB2R. CB1R is mainly distributed in the central nervous system, and previous studies have shown that activation of CB1R can be used to promote the release of neurotransmitters such as dopamine in the brain [14]. CB2R was revealed to be mainly present in the human immune system and was found to be highly expressed in bone [15, 16]. CB2R is a seven-transmembrane receptor, and in other cellular models, modulation of CB2 was found to be involved in the regulation of biological behaviors such as cell proliferation, differentiation, and migration [17]. Compared to CB1R, CB2R is present at higher levels in bone and plays an essential role in maintaining the balance between osteoclastic resorption and osteoblastic formation [18–20]. The positive effects of CB2R stimulation on osteoblast proliferation and function are generally accepted. In contrast, the stimulatory effect of CB2R on osteoclasts remains controversial in the current literature. Some studies have shown that CB2R stimulation increases the RANKL/OPG ratio, thereby favoring osteoclast formation, while others have shown the opposite [21].

In our study, we utilized Ti particles in an in vitro setting to intervene with RAW264.7 cells, aiming to simulate the impact of wear particles on macrophages under osteolysis conditions. During Ti particles-stimulated osteoclast differentiation, we observed a significant activation of osteoclast differentiation, a large intracellular production of ROS, and a significant increase in CB2 expression. To balance intracellular CB2 expression, we tried to inhibit CB2 to observe the effect on osteoclastic differentiation. The NADPH oxidase (NOX) family plays a crucial role in regulating ROS production. To investigate the specific effects, we employed a combination of a NOX1 inhibitor (ML171) and CB2 siRNA. Through our experiments, we will identify the effects that regulate the action of CB2 on osteoclastic differentiation and deepen the possible regulatory mechanisms of wear particles on osteoclastic activation during the progression of PPO.

## Result

### Ti particles promoted the production of ROS and downregulated antioxidant proteins in macrophages

**Fig. 1:**
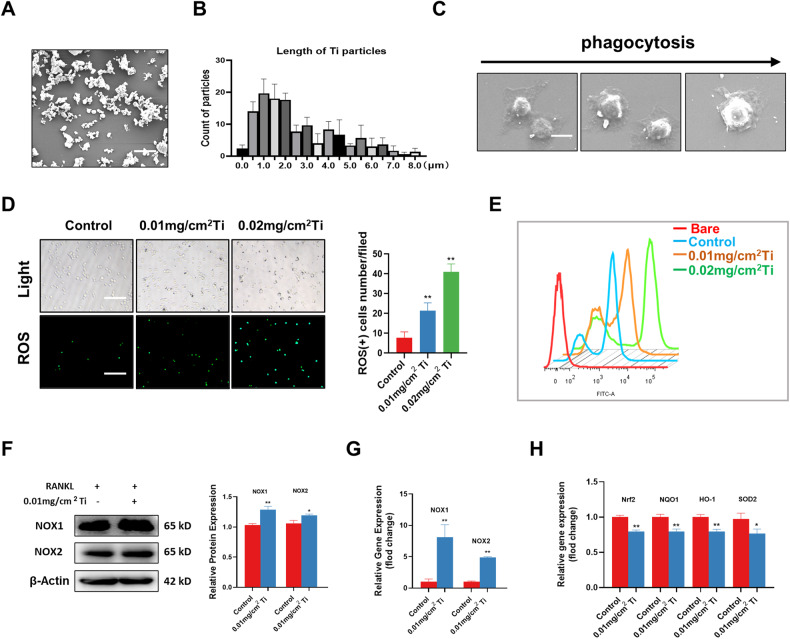
Ti particles promoted the production of ROS and downregulated antioxidant proteins in macrophages. **A** Electron micrographs of the purchased Ti particles. **B** Size analysis of Ti particles. **C** The whole process of Ti particles being phagocytosed into RAW264.7 cells under electron microscopy. **D** Intracellular ROS immunofluorescence staining and ROS(+) RAW264.7 cell counts after intervention of RAW264.7 cells with different concentrations of Ti particles. Scale bars, 100 µm. Statistical significance was determined by a one-way ANOVA with Tukey’s multiple-comparison test. **E** Intracellular ROS levels were detected by flow cytometry. **F** The expression and quantitative analysis of NOX1 and NOX2 were detected by western blotting after treatment with RANKL and Ti particles. **G** qRT-PCR analysis of the mRNA expression levels of NOX1 and NOX2. **H** qRT-PCR analysis of the mRNA expression levels of Nrf2, NQO1, HO-1, and SOD2. The data were represented as mean ± SD of three independent experiments. Statistical significance was determined by Student’s *t* test. *P < 0.05, **P < 0.01.

SEM was employed to analyze the morphology and size of the Ti particles. Our findings indicated that the Ti particles exhibited a consistent morphology and were predominantly distributed within the range of 0.5 μm to 4 μm in size (Fig. 1A, B). In vitro, we intervened with Ti particles in RAW264.7, and we observed that the Ti particles were gradually phagocytosed into the cells over time (Fig. 1C). To investigate the cytotoxic impact of Ti particles on RAW264.7 cells, we employed the CCK-8 kit to assess their influence on cell viability over a 24-h period. The findings revealed that concentrations of Ti particles below 0.02 mg/cm^2^ had a negligible effect on cell viability compared to the untreated group (Fig. S1). Then, we treated RAW264.7 cells with 0.01 and 0.02 mg/cm^2^ Ti particles to mimic the effect of Ti particles in contact with macrophages in vitro. Cellular immunofluorescence staining revealed a significant upregulation of ROS expression in RAW 264.7 cells (Fig. 1D), and flow cytometry results further confirmed this finding (Fig. 1E). Subsequently, we examined the effects of Ti particles on oxidative stress-related factors. Western blot and RT-PCR results indicated that Ti particles promoted the upregulation of NOX1 and NOX2, which are two key proteins that induce ROS (Fig. 1F, G). RT-PCR results suggested that Ti inhibited the expression of Nrf2, NQO1, HO-1, and SOD2 as well (Fig. 1H).

### Ti particles promoted the activation of osteoclasts and the formation of superoxide in mitochondria

**Fig. 2:**
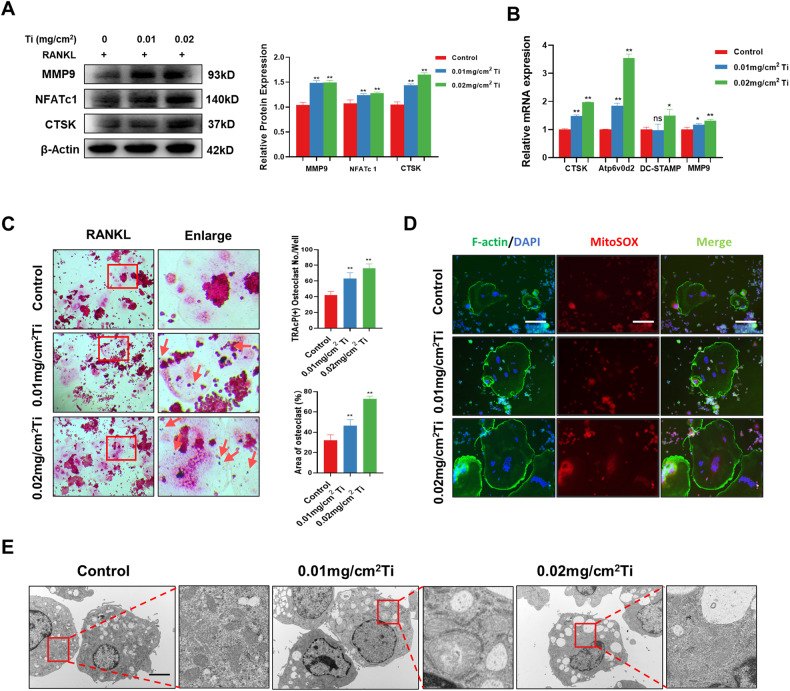
Ti particles promoted the activation of osteoclasts and the formation of superoxide in mitochondria. **A** The expression and quantitative analysis of MMP9, NFATc1, and CTSK were detected by western blotting after treatment with RANKL and Ti particles. **B** qRT-PCR analysis of the mRNA expression levels of CTSK, Atp6v0d2, DC-STAMP, and MMP9. **C** TRAcP staining and quantitative analysis of the number of positive osteoclasts and the percentage of osteoclasts in the area. Scale bars, 100 µm. **D** The expression of superoxide in osteoclasts was detected by MitoSOX immunofluorescence staining. Scale bars, 100 µm. **E** The microstructure under TEM after intervention with Ti particles. Scale bars, 2 µm. The data were represented as mean ± SD of three independent experiments. Statistical significance was determined by a one-way ANOVA with Tukey’s multiple-comparison test. *P < 0.05, **P < 0.01.

Subsequently, we induced osteoclast differentiation by treating RAW264.7 cells with a combination of 50 ng/ml RANKL and Ti particles. Four days after the intervention, we examined the expression of the intracellular osteoclast-associated marker proteins MMP9, NFATc1, and CTSK. Western blot indicated that the expression of MMP9, NFATc1 and CTSK increased compared with that in the control group (Fig. 2A). In addition, RT-PCR results indicated that Ti particles promoted the expression of the osteoclast-related genes CTSK, Atp6v0d2, DC-STAMP and MMP9 (Fig. 2B). Furthermore, TRAcP staining suggested that Ti particles facilitated the activation of osteoclasts in a concentration-dependent manner (Fig. 2C). Considering that Ti particles promoted the production of intracellular ROS in macrophages, we then further examined whether Ti particles affected the formation of oxidative substances in osteoclasts. The fluorescence staining results suggested that Ti particles promoted the formation of mitochondrial superoxide in osteoclasts (Fig. 2D). Given that one of the main sources of ROS is the respiratory chain of the inner mitochondrial membrane, we further observed the structural features of the mitochondria in the cells by TEM. We observed that the structure of mitochondria in RAW264.7 cells became slightly swollen and dim after Ti particles intervention (Fig. 2E).

### Inhibition of CB2 alleviated Ti particles-stimulated osteoclast activation

**Fig. 3:**
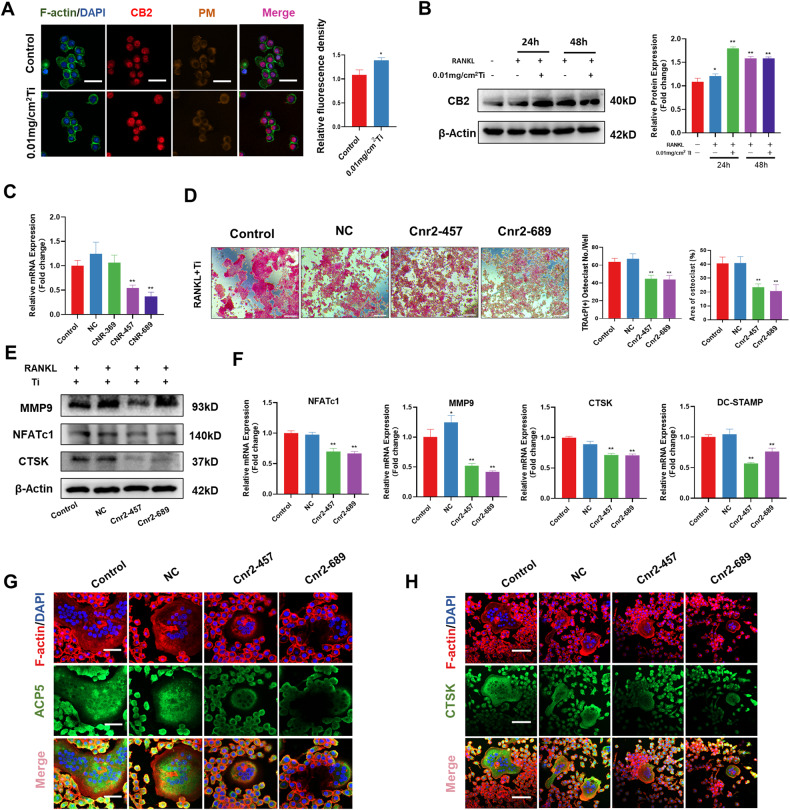
Inhibition of CB2 alleviated Ti particles-stimulated osteoclast activation. **A** Immunofluorescence assay was used to detect the expression and fluorescence quantification of CB2 under the intervention of Ti particles. Statistical significance was determined by Student’s *t* test. **B** Western blot assay and relative quantification of CB2 protein after intervention with Ti particles and RANKL. **C** RT-PCR was used to detect the effect of siRNA intervention on CB2 expression in RAW264.7. **D** Representative TRAcP staining was used to detect the effects of Cnr2-457 and Cnr2-689 on osteoclast differentiation and quantitative analysis of the number and area of osteoclasts per well. Scale bars, 200 µm. **E** Western blot revealed the effects of Cnr2-457 and Cnr2-689 on the expression of MMP9, NFATc1, and CTSK during osteoclast differentiation stimulated by Ti particles. **F** RT-PCR revealed the effects of Cnr2-457 and Cnr2-689 on osteoclast-related genes during osteoclast differentiation stimulated by Ti particles. **G**, **H** Immunofluorescence staining revealed the effects of Cnr2-457 and Cnr2-689 on the expression of ACP5 and CTSK. The data were represented as mean ± SD of three independent experiments. Statistical significance was determined by a one-way ANOVA with Tukey’s multiple-comparison test. *P < 0.05, **P < 0.01.

Cellular immunofluorescence assays revealed an increase in the fluorescence intensity of CB2 in the Ti particles group compared to the control group (Fig. 3A). Subsequently, we further examined the expression of CB2 during osteoclast differentiation. We measured the intracellular CB2 expression at the protein level during the intervention. We set up intervention groups with or without Ti particles and RANKL. Additionally, we set up groups at two-time points. The Western blot results demonstrated that the expression level of CB2 was higher in the Ti particle group (1.58 ± 0.04 vs 1.09 ± 0.08) compared to the control group after a two-day intervention with Ti particles and RANKL (Fig. 3B).

Since Ti particles promoted intracellular CB2 expression, we speculated that CB2 plays an important role in Ti particles-stimulated osteoclast differentiation. To balance intracellular CB2 expression, we interfered with this process using several CB2 siRNAs. RT-PCR confirmed that Cnr2-457 and Cnr2-689 significantly inhibited CB2 expression in RAW264.7 cells (Fig. 3C), and the western blot results further confirmed this finding (Fig. S2). Subsequently, we used Cnr2-457 and Cnr2-689 to interfere with Ti particles-stimulated osteoclast activation, we found that Cnr2-457 and Cnr2-689 inhibited osteoclast formation (Fig. 3D). Western blot analysis revealed that these two siRNAs suppressed the expression of MMP9, NFATc1 and CTSK (Figs. 3E and S3). RT-PCR results also showed that CB2 blockade inhibited expression of the osteoclast-related genes NFATc1, MMP9, CTSK, and DC-STAMP (Fig. 3F). Furthermore, the changes in ACP5 and CTSK expression during osteoclast differentiation were detected by immunofluorescence staining, and the inhibition of CB2 also suppressed the expression of ACP5 and CTSK in osteoclasts (Fig. 3G, H). Furthermore, we employed AM630, a CB2 inhibitor, to interfere the osteoclatogenesis stimulated by Ti particles. The inhibition of CB2 was further confirmed by TRAcP staining, which demonstrated a significant decrease in osteoclast activation (Fig. S4).

### Inhibition of CB2 alleviated osteoclast activation through NOX1-mediated oxidative stress signaling

**Fig. 4:**
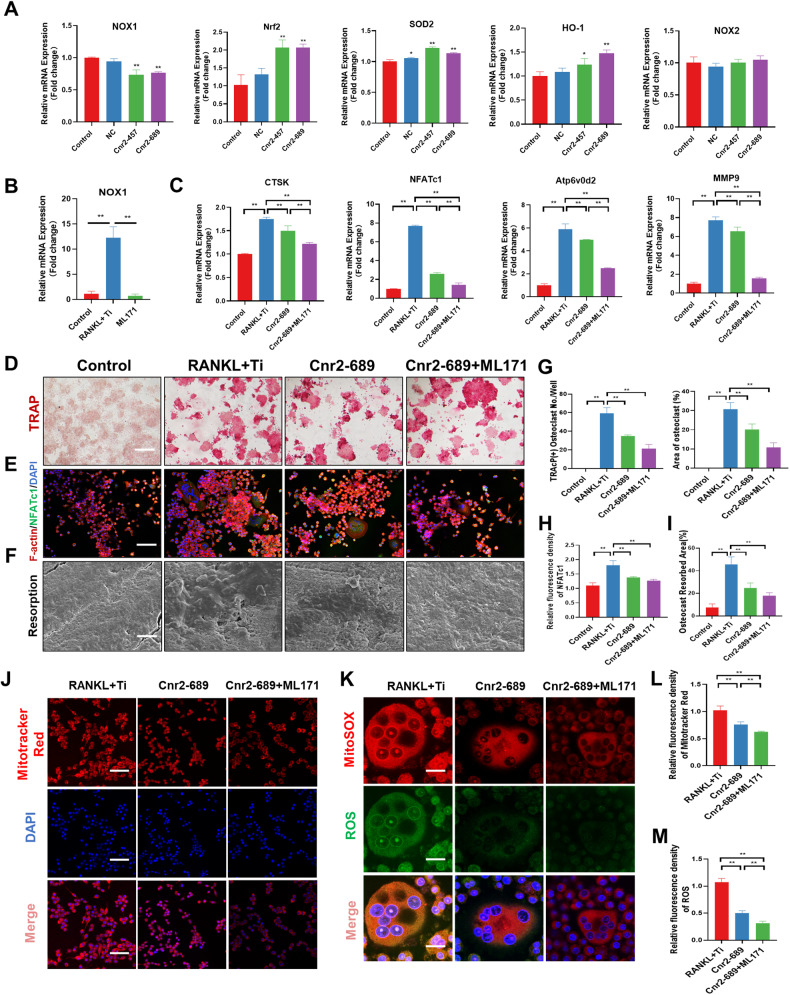
Inhibition of CB2 alleviated osteoclast activation through NOX1-mediated oxidative stress signaling. **A** RT-PCR analysis of the mRNA expression levels of NOX1, Nrf2, SOD2, HO-1, and NOX2. **B** RT-PCR was used to detect the inhibitory effect of ML171 intervention on NOX1 expression in RAW264.7. **C** RT-PCR analysis of the mRNA expression levels of CTSK, NFATc1, Atp6v0d2, and MMP9. **D**, **G** Representative TRAcP staining and quantitative analysis were used to detect the effects of Cnr2-689 and ML171 on osteoclast differentiation. **E**, **H** Immunofluorescence staining revealed the effects of Cnr2-689 and ML171 on the expression of NFATc1. **F**, **I** SEM images of the bone resorption area. **J**, **L** Immunofluorescence staining and quantitative analysis of MitoTracker Red. **K**, **M** Immunofluorescence staining and quantitative analysis of ROS. The data were represented as mean ± SD of three independent experiments. Statistical significance was determined by a one-way ANOVA with Tukey’s multiple-comparison test. *P < 0.05, **P < 0.01.

Given that the osteoclast differentiation process stimulated by Ti particles is associated with alterations in cellular oxidative stress signals, we proceeded to investigate the potential impact of inhibiting CB2 on these signals. RT-PCR analysis revealed that CB2 blockade reduced the expression level of NOX1 in cells stimulated by Ti particles. Additionally, it enhanced the expression of Nrf2, SOD2, and HO-1. However, the expression level of the NOX2 gene did not show any significant change after the Cnr2-457 (1.00 ± 0.05 vs 1.00 ± 0.09) and Cnr2-689 (1.05 ± 0.06 vs 1.00 ± 0.09) interventions, compared to the control group (Fig. 4A).

Subsequently, ML171, an inhibitor of NOX1, was used to confirm its effect on CB2-mediated regulation of osteoclast differentiation. First, CCK-8 test was employed to assess the proliferation and toxicity effects of ML171 on RAW264.7 cells (Fig. S5). We found that ML171 had little effect on RAW264.7 at 0-20 μM, and we further found that ML171 significantly inhibited the expression of NOX1 stimulated by Ti particles (Fig. 4B). We utilized a concentration of 20 μM ML171 to investigate its impact on osteoclast differentiation regulated by Cnr2-689. Our findings revealed that while Cnr2-689 alone inhibited the expression of osteoclast-related markers such as CTSK, NFATc1, Atp6v0d2, and MMP9, the combination of Cnr2-689 and ML171 further down-regulated the expression of these osteoclast-related genes (Fig. 4C). TRAcP staining revealed that when Cnr2-689 was combined with ML171, the number of osteoclasts stimulated by Ti particles was further downregulated, which was consistent with the results of NFATc1 immunofluorescence staining of osteoclasts (Fig. 4D, E, G, H). On the surface of the bone slices, we found that CB2 blockade alleviated resorption, and silencing NOX1 promotes the inhibition of bone resorption (Fig. 4F, I). Subsequently, we detected changes in membrane potential, and we found that the membrane potential of RAW264.7 was decreased after transfection with Cnr2-689, and ML171 further inhibited the mitochondrial membrane potential of RAW264.7 (Fig. 4J, L). We utilized Ti particles and RANKL to stimulate the generation of osteoclasts. Through fluorescent staining of MitoSOX and ROS, we observed that ML171 showed additional inhibitory effects on ROS expression after Cnr2-689 treatment (Fig. 4K, M).

### Inhibition of CB2 alleviated cranial bone loss in osteolytic mice

**Fig. 5:**
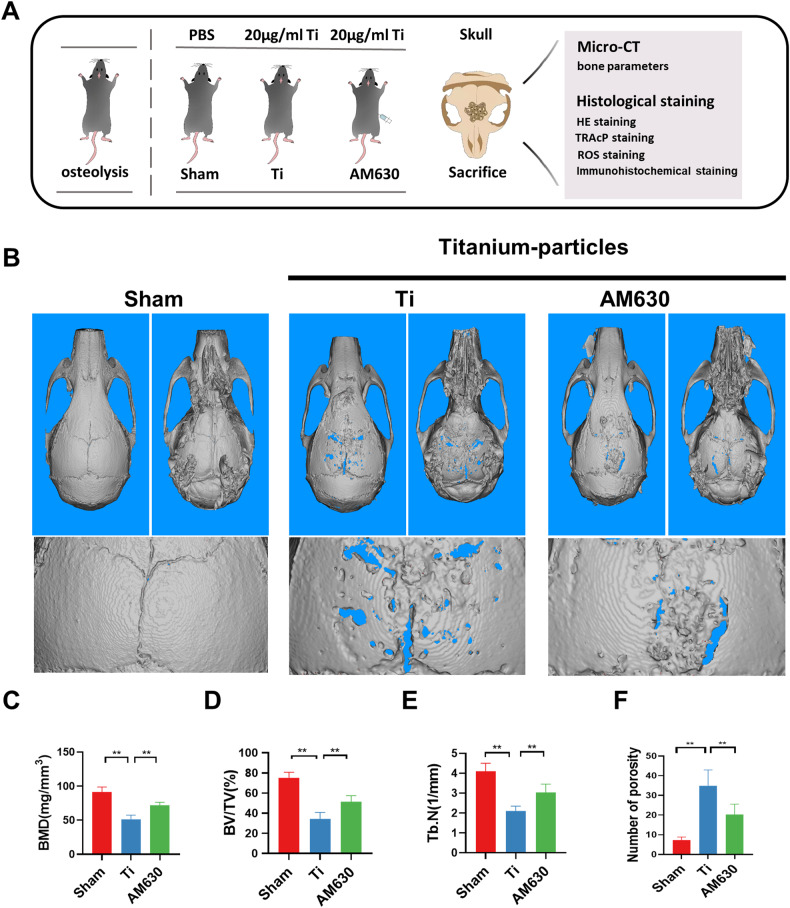
Inhibition of CB2 alleviated cranial bone loss in osteolytic mice. **A** Schematic diagram of animal modeling intervention. **B** Representative 3D and 2D reconstruction images of micro-CT. **C**–**F** Related bone parameters. *n* = 7 per group. The data were represented as mean ± SD. Statistical significance was determined by a one-way ANOVA with Tukey’s multiple-comparison test. *P < 0.05, **P < 0.01.

Our previous studies have revealed that CB2 regulates oxidative stress levels and osteoclast differentiation in vitro. Furthermore, we evaluated the specific benefits of a CB2 inhibitor (AM630) in the treatment of Ti particles-induced osteolysis in animal experiments. Figure 5A presents an overview of the experimental grouping and the procedures involved in handling the animal specimens. Following the experimental methods, we conducted micro-CT scanning and 3D reconstruction on the skulls of mice obtained from each group. Compared to the sham group, mice treated with Ti particles exhibited a significant increase in dissolved pores at the cranial top. Additionally, the local tissue displayed unevenness. After treatment with AM630, the occurrence of osteolysis at the top of the mouse skull was effectively inhibited (Fig. 5B). Bone parameter analysis results demonstrated that BMD (mg/mm^3^), BV/TV (%), and Tb. *N* (1/mm) of the Ti particles group mice was significantly decreased, and the number of porosity increased. After treatment with AM630, these parameters were effectively reversed (Fig. 5C–F).

### Inhibition of CB2 inhibited the number of osteoclasts and ROS levels in osteolysis mice

**Fig. 6:**
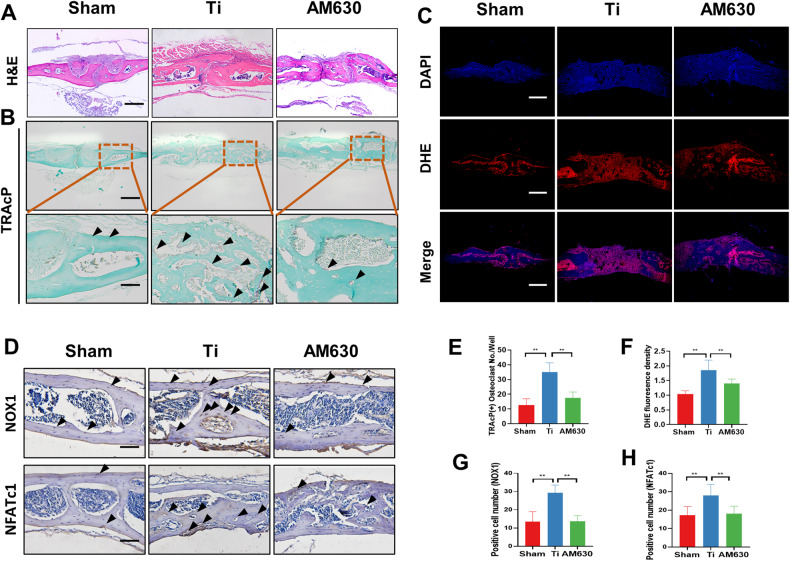
Inhibition of CB2 inhibited the number of osteoclasts and ROS levels in osteolytic mice. **A** H&E staining of skulls, Scale bar = 100 µm. **B**, **E** TRAcP staining of the skull and quantitative analysis of osteoclasts in each group of mice. Scale bar = 100 µm. **C**, **F** ROS staining and quantitative analysis of immunofluorescence staining in each group of mice, Scale bar = 100 µm. **D**, **G**, **H** Immunohistochemical staining and quantitative analysis of NOX1 and NFATc1 in decalcified bone sections. *n* = 7 per group. The data were represented as mean ± SD. Statistical significance was determined by a one-way ANOVA with Tukey’s multiple-comparison test. *P < 0.05, **P < 0.01.

To further evaluate the mechanism of AM630 in vivo, we examined the cranial osteoclasts in each group of mice. H&E staining revealed bone erosion in the cranium of mice in the Ti particles group. However, statistical analysis of the thickness of the cranial parietal bone was found to be higher in the AM630 group compared to the Ti particles group (Fig. 6A). Our findings revealed a significant increase in cranial osteoclasts in the Ti particles intervention group compared to the sham group. Additionally, a higher number of osteoclasts were observed around the damaged bone tissue. However, this phenomenon was significantly inhibited after treatment with AM630 (Fig. 6B, E). Furthermore, we detected ROS expression levels in the skull tissues. We used DHE dye for fluorescence staining. The results suggested that the expression level of ROS in the skull of mice was increased after treatment with Ti particles, and AM630 inhibited the high expression of ROS in the skull tissue (Fig. 6C, F). Similarly, immunohistochemical staining revealed that AM630 treatment inhibited NOX1 and NFATc1 signals in the skull tissue of osteolytic mice, indicating that NOX1-mediated oxidative stress signaling is an important form of CB2 action in vivo (Fig. 6D, G, H).

## Discussion

Joint diseases are a global public health problem with a high incidence and disability rate. It is definitely a difficult disease, with swollen and painful joints, bone spurs, difficulty moving and even disability [22, 23]. TJA is currently an important method for the treatment of various hip and knee lesions [24, 25]. Despite the remarkable postoperative results, there has been a noticeable rise in the occurrence of prosthesis failure attributed to PPO loosening over time.

In the initial phase after TJA, the bone volume around the prosthesis decreases dramatically and the decrease in BMD persists for a considerable period of time [26, 27]. Once a fracture is caused by long-term bone loss, revision surgery is difficult and expensive. Therefore, reducing bone loss around the prosthesis after arthroplasty is very important to prolong the use of the prosthesis and prevent periprosthetic fractures. The interface between the prosthesis and the bone is critical in determining prosthesis survival [28, 29].

Previous studies have found that large amount of wear particles continuously accumulate around the prosthesis. They induced chronic inflammation of the tissue around the prosthesis and activated osteoclast differentiation in the progression of PPO [30, 31]. The accumulation of these wear particles around the prosthesis stimulates various cells of the surrounding tissue to express cytokines and chemokines, exacerbating the chronic local inflammation and oxidative stress of the prosthesis, and ultimately leading to structural disorders in the reconstruction of the prosthesis-bone interface [32, 33].

Osteoclasts are considered to be the main functional cells in bone resorption and their overactivation has been shown to be one of the important factors contributing to skeletal destruction in vertebrates [34, 35]. Most biologically fixed joint prostheses are coated with Ti microsphere powder, and the wear particles accumulate around the joint prosthesis, causing a chronic inflammatory response. In our experiments we observed that in vitro Ti particles promoted osteoclast differentiation in a concentration-dependent manner and that this process was accompanied by a surge in intracellular ROS. ROS are derivatives of the mitochondrial respiratory chain, and studies have shown that ROS can also disrupt the balance of oxidative and antioxidant systems to activate osteoclasts [36–38]. In the late stage of osteoclast differentiation, we observed an increase in mitochondrial superoxide expression in osteoclasts. This suggested that impaired cellular oxidative systems play a significant role in the promotion of osteolysis by Ti particles. We conducted further investigations on genes associated with oxidative stress. We discovered that Ti particles had a significant impact on the expression of NOX protein, leading to its enhancement. Additionally, we observed that Ti particles also suppressed the expression of related antioxidant genes.

Cannabinoid type II receptor (CB2) is a seven-transmembrane receptor that is predominantly distributed in the immune system and has been found to be involved in bone metabolism [39]. It is composed of a typical G protein-coupled receptor. Previous studies have found that modulation of CB2 can alleviate osteoclast activity [40–42]. However, the exact mechanism is not clear. In vitro, the effects of using different agonists or inhibitors targeting CB2 that ultimately modulate osteoclastic differentiation are inconsistent. In our experiments, we constructed CB2 siRNA and found that CB2 blockade inhibited Ti particles-stimulated osteoclast differentiation. Given the observation that Ti particles enhanced osteoclast differentiation by significantly activating early levels of ROS, we conducted additional investigations to determine whether blocking CB2 inhibited osteoclast differentiation, potentially leading to altered signaling of oxidative stress.

Furthermore, we found that CB2 blockade inhibited the NOX1 signaling pathway and promoted the clearance of ROS in Ti particles-stimulated osteoclastogenesis. The suppressive effect of CB2 siRNA on osteoclastic activation was further enhanced by the inhibition of NOX1 signaling using ML171. In previous studies, NOX1-mediated ROS production has been shown to be involved in osteoclast differentiation [43, 44]. Sithole et al. discovered that the GPR120 agonist TUG-891 effectively suppressed osteoclast formation by inhibiting NOX1-mediated ROS signaling [45]. He et al. discovered that rhaponticin, a small molecule, has the potential to inhibit osteoclastic resorption. They found that it achieves this by inhibiting the expression of NOX1 and simultaneously upregulating antioxidant enzymes such as catalase, SOD-2, and HO-1 [46]. Thus, inhibition of NOX1-mediated imbalance of the antioxidant system may be potentially beneficial for the treatment of osteoclastic activating diseases.

In vivo, we used AM630 (a CB2R antagonist) in a mouse cranial osteolysis model induced by Ti particles. Our group has previously demonstrated a therapeutic effect of CB2 inhibition on osteolysis [47]. However, the exact mechanism has not been fully elucidated. In our studies, we observed a significant reduction in osteoclast as well as ROS formation in mouse cranial bone tissue in the AM630 intervention group. We also observed that AM630 inhibited NOX1 signaling in vivo, which is consistent with our in vitro data. Taken together, our results provided additional confirmation that modulation of CB2 is essential in the treatment of PPO. Moreover, our findings highlighted the significance of attenuating oxidative stress as an important intervention mechanism through which CB2 acts.

## Conclusion

Our results confirmed the efficiency of CB2 in the process of Ti particles-mediated PPO. CB2 blockade stunted Ti particles-induced osteoclast formation by oxidative stress signaling cascade. Mechanically, our research revealed that CB2 blockade modulated particles-stimulated osteoclastogenesis by downregulating NOX1 expression. Along with that, animal experiments showed that AM630 (a CB2 receptor antagonist) enhanced ROS scavenging and decreased the expression of NOX1 in osteolytic mice. Taken together, these results revealed that CB2 inhibition provides a rationale as a potential therapeutic strategy for preventing PPO.

## Methods

### Drugs and reagents

Ti particles were purchased from Alfa Aesar (Ward Hill, MA). Recombinant RANKL was obtained from R&D Systems (Minneapolis, USA). RAW264.7 cells were obtained from the Cell Bank of the Chinese Academy of Sciences (Shanghai, China). RNA interference sequences were obtained from GenePharma (Suzhou, China). DMEM/high glucose and fetal bovine serum (FBS) were bought from Thermo Fisher Scientific (St. Louis, MO, USA). ML171 was bought from the Master of Bioactive Molecules (Suzhou, China). AM630 was bought from Beyotime (Shanghai, China). The primary antibodies employed in our experiment included CTSK (A5871, ABclonal, Wuhan, China), NFATc1 (A1539, ABclonal), MMP9 (A0289, ABclonal), ACP5 (A2528, ABclonal), NOX1 (A8527, ABclonal), NOX2 (A19701, ABclonal) and β-Actin (AC006, ABclonal). The ROS Assay Kit was bought from Beyotime (Shanghai, China).

### Processing of Ti particles

Ti particles were first treated at 180 °C for 12 h and soaked in ethanol for 2 days to remove residual endotoxin. The properties of the Ti particles were evaluated by scanning electron microscopy (SEM). RAW264.7 cells were intervened with Ti particles and the morphological characteristics of the cells were observed under SEM.

### Intracellular ROS assay

After treating RAW264.7 cells with Ti particles or other pharmacological interventions, the samples were rinsed with serum-free DMEM medium. Rosup, a ROS-positive control used to enhance the detection of intracellular ROS, was diluted with medium at a concentration of 1:1000. The samples were then treated with diluted Rosup for 30 min, protected from light, and washed again three times with serum-free medium. Subsequently, DCFH-DA dye was prepared and the cell samples were incubated at 37 °C for 30 min, followed by three additional washes of ~5 min each. Finally, the treated RAW264.7 cells were either observed using an inverted microscope (Zeiss, Germany) to count the number of positive ROS cells or analyzed using a flow cytometer to measure the expression level of ROS.

### In vitro osteoclastogenesis assay

RAW264.7 cells were digested and counted. The samples were pretreated with basal medium containing 0.01 and 0.02 mg/cm^2^ of Ti particles in 48-well plates. Each well was inoculated with 15,000 cells. Following this, 50 ng/ml RANKL was added to induce osteoclasts, as described in previous literature reports [48–50]. The cells were placed in the incubator and continued to be cultured for four days, with culture medium changes every 2 days until osteoclast formation.

### TRAcP staining

RAW264.7 cells were treated with medium containing RANKL and either 0.01 or 0.02 mg/cm^2^ of Ti particles. After osteoclast formation, the cells were washed with phosphate buffer solution (PBS) and then fixed by aspirating 200 µl of paraformaldehyde (Beyotime, China) into the plate for 15 min. After washing, the TRAcP kit (Bizhong Bio, Suzhou) was used and staining solution was prepared according to the instruction manual. TRAcP staining solution was added to the experimental cells and discarded after 30 min of staining. The number of osteoclasts and the area occupied by osteoclasts in each group were counted under the microscope using ImageJ (Bethesda, USA).

### Western blot

RAW264.7 cells were collected according to the experimental purpose. Then, samples were lysed using RIPA buffer to harvest the total protein on ice. Samples were quantified by using a BCA protein kit (Beyotime, China) and added to electrophoresis buffer (Beyotime, China). The voltage was set to 60 V. After 15 min when the samples were completely in the lower separation gel, the voltage was increased to 120 V. When the electrophoresis was completed, the finished gel was fixed on the treated PVDF membrane. Suitable protein bands were excised from the transferred PVDF according to the desired molecular weight. Diluted primary antibody solution was added and shaken overnight at 4 °C to stabilize the primary antibody. Horseradish peroxidase-conjugated secondary antibodies (Beyotime, China) were diluted and incubated at room temperature for another 1 h. ECL reagents (NCM Biotech, China) were employed to assay antibody-antigen complexes. After exposure and obtaining protein blot images, the ImageJ (Bethesda, USA) was used to quantify the protein density.

### Quantitative RT-PCR (qRT-PCR)

Total RNA of samples was extracted by lysis with TRIzol reagent (Thermo Fisher Scientific, USA) and subsequent extraction with chloroform. The upper clear liquid layer containing RNA was extracted and an equal volume of isopropanol was added. Subsequently, the RNA was reverse transcribed to cDNA using a thermal cycler (Thermo Fisher Scientific, USA). A mixture of TB Green reagent (TaKaRa, Japan), RNase-free H_2_O (TaKaRa, Japan) and the forward and reverse primers listed in Table S1 were used. LightCycler® 480 (Roche Diagnostics International Ltd., Switzerland) was employed to measure the gene levels of CTSK, MMP9, NFATc1, Atp6vod2, DC-STAMP, NOX1, NOX2, Nrf2, NQO1, HO-1 and SOD2. Among them, the GAPDH gene was used as a housekeeping gene and the fold change was calculated based on the comparative 2^−ΔΔCq^ method.

### Transmission electron microscopy (TEM)

TEM was employed to observe the mitochondrial structures of RAW264.7 cells. Initially, the cells were treated with 0.01 and 0.02 mg/cm^2^ of Ti particles, followed by collection, centrifugation, washing, and fixation with 2.5% glutaraldehyde. Subsequently, the samples were dehydrated using gradient concentrations of ethanol. The treated cells were then embedded in resin and sectioned. Finally, the intracellular structures were examined using TEM (Hitachi H-7650, Japan).

### Bone resorption assay

RAW264.7 cells (2 × 10^4^) were plated on bovine cortical bone slices (JoyTech Biotechnology, China) in 48-well plates, and cultured with Ti particles, 50 ng/ml RANKL, and other pharmacological interventions for 6 days to stimulate osteoclasts. Bone resorption pits were imaged using an FEI Quanta 250 scanning electron microscope (Hillsboro, USA). ImageJ was used to analyze four areas in bone slices from the bone resorption assay.

### Cell transfection

RAW264.7 cells were transfected with siRNA and Lipofectamine 3000 (Thermo Fisher Scientific, USA). The sequences of the CB2 transfection reagent are shown in Table S2. More specifically, we cultured RAW264.7 cells in six-well plates and strictly controlled the number of cells to ensure that the density of cells is maintained at about 60% when transfected. The appropriate amount of siRNA to be transfected was added to the cell culture medium containing no serum according to the manufacturer’s requirements. Subsequently, we added Lipofectamine 3000 at room temperature for 20 min to form a stable transfection complex. The cells were then replaced with fresh medium, and the appropriate amount of transfection complex was added. After treatment for 48 h, we collected samples for subsequent experiments. The expression of CB2 in the transfected cells was measured by western blot or RT-PCR to confirm its effectiveness.

### Cell proliferation and viability assay

To assess the cytotoxicity of Ti particles and ML171, we utilized a CCK-8 assay kit (ApexBio, Boston, USA). The samples were planted in 96-well plates at a density of 5000 cells per well. They were then treated with Ti particles or ML171 and incubated for 1 day. Afterward, the cells were incubated with DMEM containing 10% CCK-8 solution for 3 h at 37 °C, while being strictly protected from light. Finally, we assessed the absorbance at 450 nm using a microplate reader (BioTek, USA).

### Mitochondrial membrane potential assay

We measured the mitochondrial membrane potential using MitoTracker Red CMXRos (Beyotime, China), a red fluorescent dye used to stain the mitochondria of living cells. In brief, cells were incubated with 50 nM MitoTracker Red CMXRos for 30 min. Subsequently, the nuclei of the samples were stained with DAPI (Beyotime, China). Images were obtained using a Zeiss laser scanning microscope (LSM510, Zeiss).

### Immunofluorescence staining

Cells were rinsed with PBS and subsequently fixed on ice with 4% paraformaldehyde solution. Then, they were permeabilized with 0.1% Triton X-100 (Beyotime, China) for 10 min at room temperature and sealed with PBS blocking solution (Beyotime, China) for 1 h. In addition, primary antibodies were added for 12 h, including anti-CB2, anti-NFATc1, anti-CTSK, and anti-ACP5. Finally, the samples were washed with PBS for 15 min and incubated with secondary antibodies (Alexa Fluor® 488 or Alexa Fluor® 647, Abcam, UK) and phalloidin iFluor™ (Yeasen, China). We placed the coverslips in a fluorescent antifade solution containing DAPI (Beyotime, China). Microscopic images were obtained by a Zeiss laser scanning microscope (LSM510, Zeiss).

### MitoSOX immunofluorescence staining

For MitoSOX staining of osteoclasts, the cells were incubated for 1 h with MitoSOX solution (Thermo Fisher Scientific, USA) according to the manufacturer’s instructions. DAPI stain (Beyotime, China) was applied to restain the nuclei. Microscopic images were obtained by a Zeiss laser scanning microscope (LSM510, Zeiss).

### Animals and Ti particles-induced osteolysis model

The animal experiments in this study were approved by the Ethics Committee of the Soochow University. Twenty-one 8-week-old C57/BL male mice were used in our experiment. The animals were kept in a basic environment: room temperature of about 25 °C, humidity controlled at 60%, 12 h alternating day and night and regular ventilation. The experimental animals were divided into three groups: the sham group (sham operation + PBS treatment), Ti group (Ti particles operation + PBS treatment) and AM630 group (Ti particles operation + AM630 injection). Ti particles were sterilized to remove the surface endotoxin, rinsed repeatedly with PBS and stored at 4 °C. Mice were randomly assigned to experimental groups, and no data were excluded. The investigators were not blinded to group allocation during experiments. For Ti particles operation, the mouse head was debrided and disinfected with alcohol. Subsequently, a 0.6 cm incision was carefully performed on the scalp, followed by the injection of a preconfigured solution consisting of Ti particles. The incision was tightly sutured, and the wound was disinfected. In the treatment group, mice were injected with 200 μg/kg AM630 for four weeks (three times a week until execution).

### Micro-CT analysis

The craniums of each group of mice were collected and placed in 10% neutral formalin for 2 days, followed by scanning with a high-resolution micro-CT scanner (SkyScan1176, Belgium). The machine parameters were set to 9 μm resolution, 0.7° rotation angle, and 170 mA current. After scanning, the scanning system was turned off and the acquired data were reconstructed in three dimensions using NRecon and SkyScanRECON software. The bone mineral density (BMD), bone volume/tissue volume (BV/TV), trabecular number (Tb. N, 1/mm) and number of pores were quantified.

### Histological staining and analysis

The craniums collected from each group were meticulously trimmed using surgical instruments to remove the surrounding soft tissue and ensure thorough decalcification. Subsequently, the specimens were embedded and sectioned.

For H&E staining, the sections were baked at 60 °C for 40 min and then further dewaxed in xylene. After dewaxing, gradient ethanol dehydration was performed. Tissue sections were stained with hematoxylin and restained with eosin (Beijing Leagene Biotechnology, China). The stained sections were immersed in gradient ethanol, dried in a fume hood and sealed with resin. For TRAcP staining, TRAcP solution (Bizhong Bio, Suzhou) was prepared according to the manufacturer’s instructions. After the sections were dewaxed and dehydrated, they were incubated in a 37°C incubator for 1 hour. The slices were then washed with PBS for 5 min and soaked in xylene for 10 min. For immunohistochemistry, primary antibodies against NFATc1 (ab25916, Abcam) and NOX1 (ab55831, Abcam) were employed. Photographs were taken with an Axiovert 40 C optical microscope (Zeiss, Germany), and the positive cells in the area were counted and quantified by ImageJ software (Bethesda, USA).

### Dihydroethidium (DHE) staining

After the slices were dehydrated, DHE staining (Beyotime, China) was prepared according to the instructions and incubated. After incubation, the DAPI dye was incubated for 10 min to restain the nuclei. Eventually, the fluorescence intensity of each group of stained areas was observed under an inverted fluorescence microscope (Axiovert 40 °C, Zeiss, Germany).

### Statistical analysis

The resultant data were analyzed using GraphPad Prism 8.0 software, and the mean ± standard deviation was used for data results that conformed to a normal distribution. No statistical methods were used to predetermine the sample size. For statistical analysis between two groups, Student’s *t* test was used. For data in three or more groups, one-way analysis of variance post was used. A *P* value < 0.05 was considered significant.

### Supplementary information

Supplementary materials

Raw Western Blot

## Data Availability

The datasets generated and/or analyzed during the current study are not publicly available but are available from the corresponding author on reasonable request. The original western blot for this study can be found in the Supplementary Information File.
