# Supplementary material for: CB2 regulates oxidative stress and osteoclastogenesis through NOX1-dependent signaling pathway in titanium particle-induced osteolysis
Source: Cell Death Discov. 2023 Dec 16;9:461. doi: 10.1038/s41420-023-01761-y (PMC10725463; doi:10.1038/s41420-023-01761-y)
Supplement: Supplementary file 1 — Supplementary materials [file 41420_2023_1761_MOESM1_ESM.docx]

**Title Page**

**Title of the article**

CB2 regulates oxidative stress and osteoclastogenesis through NOX1-depedent signaling pathway in titanium particle-induced osteolysis

**Running Title:** CB2 regulates osteolysis via NOX1 pathway

**The name(s) of the author(s)**

Huaqiang Tao, MS ^1, a^; Xueyan Li, MS ^2, a^; Miao Chu, MS ^1, a^; Qiufei Wang, MS ^3^; Ping Li, MS ^4^; Qibin Han, MS ^5^; Kai Chen, MS ^1^; Pengfei Zhu, MS ^1^; Yuefeng Hao, PhD ^5^; Xing Yang, PhD ^5, *^; Dechun Geng, PhD ^1, *^; Ye Gu, PhD ^3, *^.

**Name and address of the institution**

^1^ Department of Orthopedics, The First Affiliated Hospital of Soochow University, No. 188 Shizi Street, Suzhou, Jiangsu, China.

^2^ Anesthesiology department, Suzhou Municipal Hospital, Nanjing Medical University Affiliated Suzhou Hospital, 242, Guangji Road, Suzhou, Jiangsu, China.

^3^ Department of Orthopedics, Changshu Hospital Affiliated to Soochow University, First People’s Hospital of Changshu City, the First Affiliated Hospital of Soochow University, Suzhou, Jiangsu, China.

^4^ Department of Central Laboratory, Nanjing Medical University Affiliated Suzhou Hospital, Gusu School, Suzhou, Jiangsu, China.

^5^ Orthopedics and Sports Medicine Center, Suzhou Municipal Hospital, Nanjing Medical University Affiliated Suzhou Hospital, 242, Guangji Road, Suzhou, Jiangsu, China.

^a^ **Contribute equally to this work**

* **Correspondence and requests for materials should be addressed to**

Xing Yang Email: xingyangsz@126.com;

Dechun Geng Email: [szgengdc@suda.edu.cn](mailto:szgengdc@suda.edu.cn);

Ye Gu Email: [edwingguye@126.com](mailto:edwingguye@126.com).

**Supplementary materials.**

Table S1. Primers used in RT-PCR.

| Gene | Primer Sequence (F) | Primer Sequence (R) |
| --- | --- | --- |
| CTSK  MMP9  NFATc1  Atp6v0d2  DC-STAMP  NOX1  NOX2  Nrf2  NQO1  HO-1  SOD2 | CTTCCAATACGTGCAGCAGA  CGTGTCTGGAGATTCGACTTGA  GAGAATCGAGATCACCTCCTAC  GACCCTGTGGCACTTTTTGT  AAAACCCTTGGGCTGTTCTT  GCACAGCTTCCACACTTTC  TGGCGATCTCAGCAAAAGGT  TCTTCACTGCCCCTCATC  TTTTCCCCAGCTTGTCTG  GCAACAAGCAGAACCCA  ATGCCCAAACCTATCGTG | TCTTCAGGGCTTTCTCGTTC  TTGGAAACTCACACGCCAGA  TTGCAGCTAGGAAGTACGTCTT  GTGTTTGAGCTTGGGGAGAA  AATCATGGACGACTCCTTGG  CTCTCAGGTCGGCCAAG  TTCAGCCCCAACCAGGAAAC  CTCCTGCCAAACTTGCTC  TCCTTTTCCCATCCTCGT  GTGAGGCCCATACCAGAA  GACCCAATGAGCCAAAGA |
| GAPDH | GGTTGTCTCCTGCGACTTCA | TGGTCCAGGGTTTCTTACTCC |

Table S2. The sequence of CB2 transfection reagent.

| Gene | sense | antisense |
| --- | --- | --- |
| NC  Cnr2-369  Cnr2-457  Cnr2-689 | UUCUCCGAACGUGUCACGUTT  GAGUACAUGAUCCUGAGCATT  CCGUGCUCUAUAUUAUCCUTT  CCGCUACCUAUGUCUGUGUTT | ACGUGACACGUUCGGAGAATT  UGCUCAGGAUCAUGUACUCTT  AGGAUAAUAUAGAGCACGGTT  ACACAGACAUAGGUAGCGGTT |


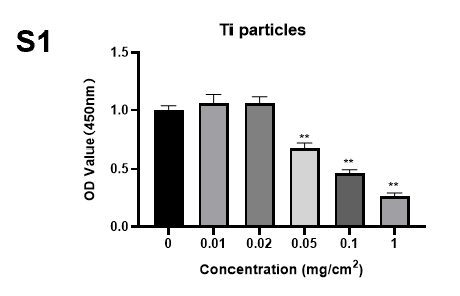


Fig. S1 Cell viability was assessed using a CCK-8 kit after incubation for 24 hours with varying concentrations of Ti particles. Statistical significance was determined by a one-way ANOVA with Tukey’s multiple-comparison test. ***P* < 0.01, compared with the 0 mg/cm^2^ group.


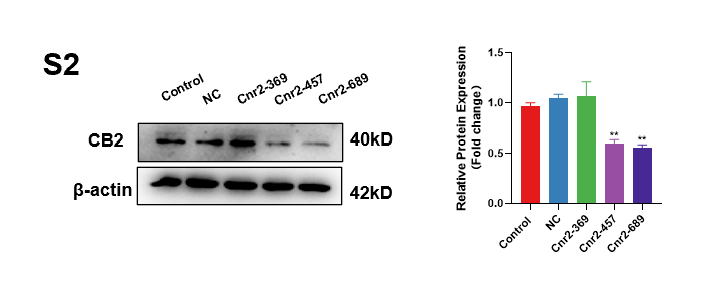


Fig. S2 Western blot revealed that validation of the effect of several CB2 siRNAs to inhibit CB2 expression. Statistical significance was determined by a one-way ANOVA with Tukey’s multiple-comparison test. n=3, ***P*<0.01.


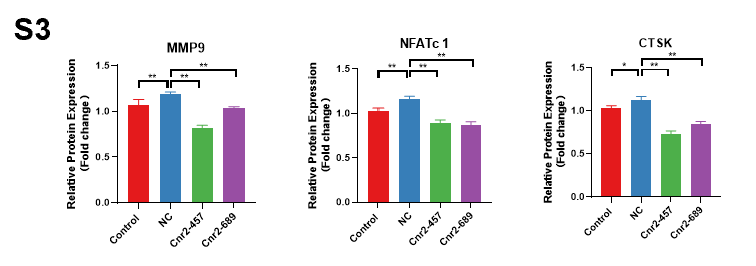


Fig. S3 Quantitative analysis of MMP9, NFATc 1 and CTSK were detected by western blot after the intervention of CB2 blockade. Statistical significance was determined by a one-way ANOVA with Tukey’s multiple-comparison test. n=3, **P*<0.05, ***P*<0.01.


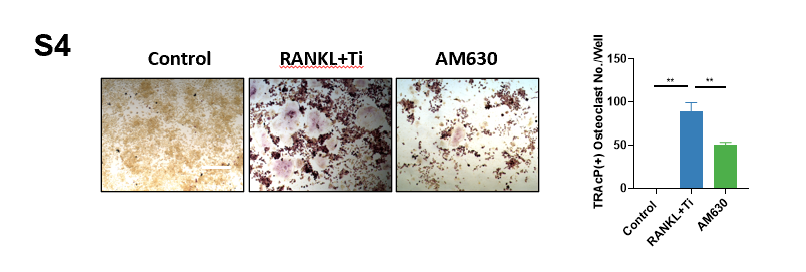


Fig. S4 TRAcP staining and quantitative analysis revealed that AM630 inhibited the number of positive osteoclasts and the percentage of osteoclasts in the area, the scale =100 μm. Statistical significance was determined by a one-way ANOVA with Tukey’s multiple-comparison test. n=3, **P*<0.05, ***P*<0.01.


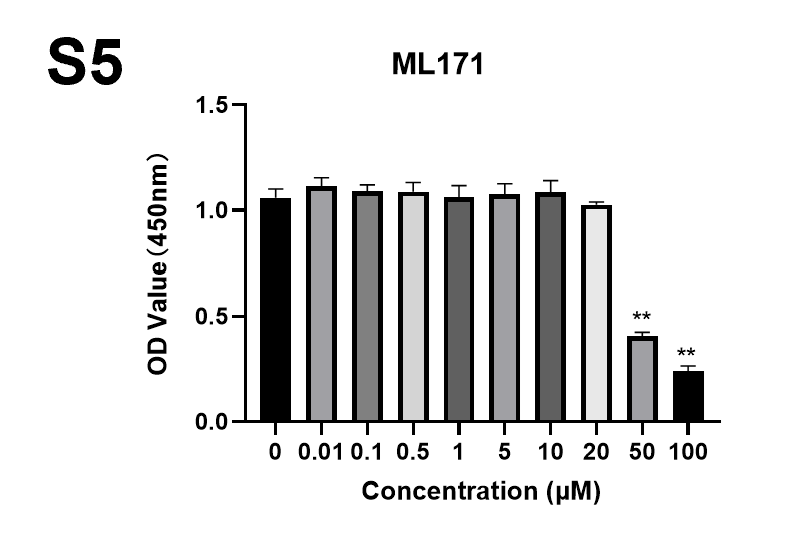


Fig. S5 Cell viability after incubation for 24 hours with different concentrations of ML171 was assessed using a CCK-8 kit. Statistical significance was determined by a one-way ANOVA with Tukey’s multiple-comparison test. n=5. ***P*< 0.01, compared with the 0 μM group.
