# Supplementary figures and images for: CB2 regulates oxidative stress and osteoclastogenesis through NOX1-dependent signaling pathway in titanium particle-induced osteolysis
Source: Cell Death Discov. 2023 Dec 16;9:461. doi: 10.1038/s41420-023-01761-y (PMC10725463; doi:10.1038/s41420-023-01761-y)

Figure 1F

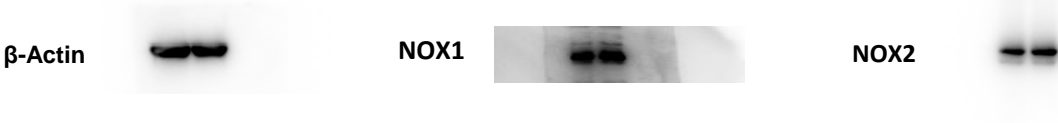

Figure 2A

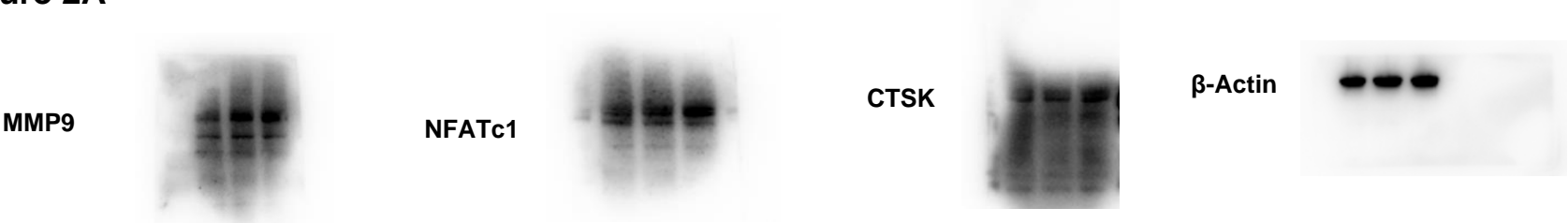

Figure 3B

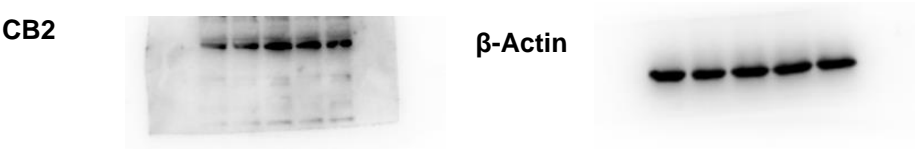

Figure 3E

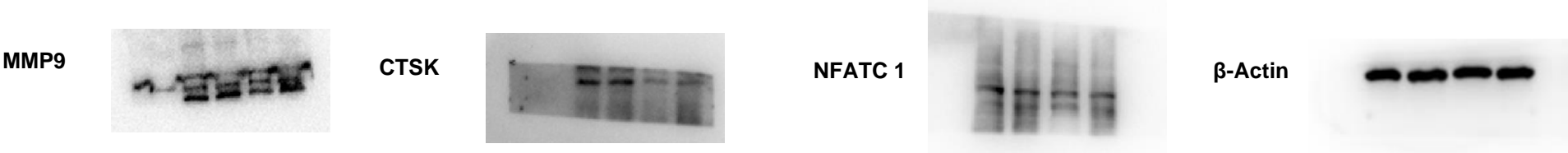

Figure S2

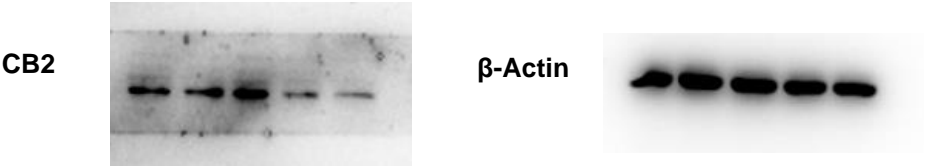

Supplement: Supplementary file 2 — Raw Western Blot [file 41420_2023_1761_MOESM2_ESM.pdf]
